# Impact of obesity and other key risk factors on adverse outcomes in COVID-19 patients in critical care settings

**DOI:** 10.12669/pjms.41.5.9302

**Published:** 2025-05

**Authors:** Mehwish Iftikhar, Amna Rizvi, Saba Zartash, Arsalan Nawaz

**Affiliations:** 1 Mehwish Iftikhar, FCPS Endocrinology, FCPS Medicine, Assistant Professor of Endocrinology, Department of Endocrinology and Metabolism. SIMS, Services Hospital Lahore, Pakistan; 2 Amna Rizvi, FCPS Endocrinology FCPS Medicine, SCE Endocrinology UK, Associate Professor Endocrinology, Department of Endocrinology and Metabolism. SIMS, Services Hospital Lahore, Pakistan; 3 Saba Zartash, FCPS Medicine, Senior Registrar of Medicine, Department of Critical Care, SIMS, Services Hospital Lahore, Pakistan; 4 Arsalan Nawaz, FCPS Endocrinology FCPS Medicine, MRCP. Assistant Professor, Department of Medicine, UCMD, University of Lahore Teaching Hospital, Lahore, Pakistan

**Keywords:** Obesity, COVID-19, Critical Care

## Abstract

**Background & Objective:**

Although the global emergency caused by COVID-19 was officially declared over in 2023, the pandemic by no means has completely disappeared. Study explored the interconnection between obesity and critical outcomes in intensive care. Focusing on the infiltration of viruses and increased risk with obesity and other factors, this study provides insights for tailored interventions. This study is devised to establish the effect of obesity and other associated factors with outcomes of patients having COVID 19 infection in the critical care setting.

**Methods:**

This observational study was performed in the COVID-19 ICU of Services Hospital Lahore from December 10, 2020 to February 10, 2021. One hundred fifty consecutive COVID-PCR positive, conscious patients with Age (18-80 years) and BMI (18 to ≥ 40) were included. Patients with inability to provide informed consent and pregnancy were excluded. BMI was categorized into non-obese (18-29.9) and obese (≥ 30), with outcomes assessed during ICU stay. Analysis involved descriptive statistics and logistic regression to predict outcomes.

**Results:**

Study showed an average age of 59.73 years, BMI 30.14 kg/m², and 80.66% baseline O2 levels. BMI’s had significant impact on COVID-19 outcomes, emphasizing its association with severity, comorbidities, ARDS, and mortality (p<0.05). Logistic regression indicates BMI, SpO2, and HCO3 significantly predict outcomes, with increased BMI elevating mortality risk, while decreased SpO2 and HCO3 increase mortality likelihood (p<0.05).

**Conclusion:**

These findings highlight considering obesity as a critical factor in COVID-19 prognosis. This research has contributed valuable insights needed to grapple with the complexities of the pandemic.

## INTRODUCTION

Top of Form The appearance of severe acute respiratory syndrome coronavirus 2 (SARS-CoV-2), initially identified as HCoV-19, in late 2019 has unleashed a global pandemic, remaking the world’s landscape.^1^ Although the global emergency was officially declared over in 2023, the pandemic by no means has completely disappeared. Recent WHO figures^2^ are 238,416 (COVID-19 Cases) only in last 28 days. Involvement is multisystemic,^3^ As an RNA virus, SARS-CoV-2 belongs to the beta coronavirus genus and shares genetic kinship with bat coronaviruses and its ancestor, SARS-CoV-1.^4^ The virus manipulates the angiotensin-converting–enzyme (ACE2) receptor to infiltrate human cells, akin to SARS-CoV-1. It Facilitates human-to-human transmission through respiratory droplets, direct contact, or touching contaminated surfaces. SARS-CoV-2 exhibits a spectrum ranging from asymptomatic carriers to severe infections with fatal complications.

Against this backdrop, the increasing global prevalence of obesity, affects nearly two billion people in 2016. It adds a convincing layer to the COVID-19 narrative. Body mass index (BMI) serves as a key metric. It designates overweight (25-30 BMI) and obesity (BMI above 30). Obesity’s intricate relationship with infection susceptibility is underscored by elevated inflammatory cytokines in obese individuals, impacting immune function.^5^ Mechanisms underlying the compromised immunity include dysfunctional B lymphocytes. This fosters systemic inflammation and a suboptimal response to vaccines.^6^

The ramifications of obesity extend to its status as a risk factor for heightened infections, with adipose tissue acting as a dynamic endocrine organ. This tissue releases bioactive molecules like adipokines and leptin, influencing immune responses. In the context of SARS-CoV-2, obesity-induced alterations in airways and the immune system assume critical significance.^7^ A French study revealed a notable association between obesity, intensive care unit admissions for SARS-CoV-2, and increased reliance on invasive mechanical ventilation^8^,

Similarly, in Shenzhen, China, COVID-19-afflicted obese individuals faced an elevated risk of severe pneumonia, particularly among men.^9^ As the world navigates the paradigm shift ushered in by the COVID-19 era, this exploration of the intricate interplay between SARS-CoV-2 and obesity aims to unravel the complexities at the intersection of infectious diseases and metabolic health, offering insights crucial for holistic patient care and public health strategies. This study was designed to establish the effect of obesity and other key factors with outcomes of patients having mild to severe COVID 19 infections in the critical care setting.

## METHODS

The observational descriptive study was conducted as investigation for six months from December 10, 2020, to February 10, 2021. in the COVID-19 Intensive Care Unit at Services Hospital Lahore. A total of 150 patients were recruited using a Consecutive Sampling technique.

Ethical Approval: The study was approved by the by the institutional review board of Services Hospital. Ref. (IRB/2020736/SIMS, dated: November 9, 2020) and registered with Clinical Trials.gov (NCT04674553).

### Inclusion Criteria:

The study enrolled COVID-19 PCR confirmed patients both male and female who presented to the ICU with age range of 18-80 years focusing on individuals exhibiting mild to severe disease. Only conscious patients at initial presentation, within a BMI in range of 18 to ≥ 40, were considered eligible for participation.

### Exclusion criteria:

Individuals unable to provide free informed consent and pregnant females were excluded, Additionally, patients with gross ascites, amputation, or other conditions that could compromise the accurate measurement of BMI were also excluded. We took BMI (kg/m²) 18-29.9 as non-obese and BMI (kg/m²) ≥ 30 as obesity.

One hundred fifty patients were included after written informed consent and their BMI was calculated on admission, using standard anthropometry equipment. Their clinical (progress of disease, need of mechanical ventilation), radiological, and baseline biochemical parameters (SpO_2_, BMI, pH, HCO_3_, TLC, Ferritin, CRP, LDH, D-Dimers) during ICU stay were recorded on a predesigned proforma. They all were given customary treatment and *outcome* was defined as whether the patient was cured and discharged or had mortality.

### Statistical Analysis:

SPSS version 25 was used for analysis. Mean with standard deviation were calculated for quantitative variables like (age, SpO_2_, BMI, pH, HCO_3_, TLC, Ferritin, CRP, LDH, D-Dimers). Frequencies were determined for qualitative variables like (Outcome, gender, comorbidities, presence of acute respiratory distress syndrome (ARDS), severity of COVID-19 on admission and mode of respiratory support given). The stratification of outcome was done with various variables. To test whether age, gender, BMI, SpO_2_, CO_2_, HCO_3_, Ferritin, LDH, CRP, TLC, D-dimer and comorbid conditions predict the outcome of patient in terms of cure or mortality, logistic regression was performed.

## RESULTS

On scrutinizing the outcomes of our study which focused on the association between obesity and COVID-19 severity, noteworthy patterns emerged. Several variables, listed along with their mean and standard deviation values are listed in Table-I. Some relevant results that can be inferred include the average age of the patients being around 59.73 years, the average BMI being 30.14 kg/m^2^, and the average O_2_ level at baseline being 80.66%. Male (56%) participants were slightly more than females, almost (17%) were severely affected with 28.7% got mortality.

**Table:** COVID Severity Criteria.

Mild	Asymptomatic, (SpO_2_) >94%, without any hemodynamic compromise or X-ray findings.
Moderate	Hypoxia, (SpO_2_) <94 % but >90% that is (93% to 90%) with or without X-ray < 50% involvement with infiltrates
Sever	SpO_2_ < 90% but >80% (80% -89%) on room air with or without an X-ray with > 50% involvement with infiltrates

**Table-I:** Demographic characteristics and Lab parameters of population under study (n=150)

Variables	Mean	Std. Deviation (SD)
Age (In completed years)	59.73	±11.35
BMI (kg/m²)	30.14	±7.30
Lab Parameters		
SpO_2_(%) at baseline	80.66	±9.15
PCO_2_ mmHg	58.46	±18.04
HCO_3_mEq/L	21.88	±5.75
pH Value at baseline	7.25	±0.14
TLC (10^9/L) at baseline	14.56	±5.68
Lymphocytes (10^9/L) at baseline	3.15	±1.50
Ferritin (mcg/L) at baseline	920.28	±403.70
LDH (IU/L) at baseline	508.09	±315.06
D-dimer (mcg/mL) at baseline	14.98	±90.15
CRP (mg/L) at baseline	121.23	±68.49
	*Numbers*	*Percentage %*
*Gender*		
Male	84	56%
Female	66	44%
*Smoking*		
Yes	27	18%
No	123	82.7%
*Severity of COVID-19*		
Sever	26	17.3%
Mild	124	82.7%
*ARDS*		
Yes	21	14%
No	129	86%
*Mechanical Ventilation*		
Yes	58	38.7%
No	92	61.3%
*Co Morbid Condition*		
Yes	50	33.3%
No	100	66.7%
*Outcome*		
Cure &Discharge	107	71.3%
Mortality	43	28.7%

* Significant at 5%

Data are numbers and percentages (%) unless indicated otherwise. P-value was significant at 0.05 The stratified analysis of the association of BMI with various outcomes in COVID-19 patients is presented in Table-II, considering different confounding variables such as age, gender, smoking status, disease severity, comorbidities and various lab parameters. It can be inferred from bivariate analysis that Outcome of COVID-19 infected individuals is affected by various factors, important being BMI, severity of disease and co morbidities as shown by *p-value* (<0.05), for example, in case of BMI, a *p-value*=0.00 suggests a strong statistical significance, indicating that the relationship between BMI and these outcomes is unlikely to be due to chance.

**Table-II:** Stratified analysis of COVID-19 outcome with different variables

Variable	Sub Groups	Outcome		Total	(*p−value)*
		Cure	Mortality		
Age	18-40 Years	7(63%)	4(36.4%)	11(100%)	(0.09)
	41-60 Years	52(80%)	13(20%)	65(100.0%)	
	61-80 Years	48(65%)	26(34.2%)	74(100.0%)	
Obesity	Non-Obese (BMI (kg/m²):18-29.9)	58(85.3%)	10(14.7%)	68(100.0%)	(0.001)*
	Obese (BMI (kg/m²): ≥30)	49(59.8%)	33(40.2%)	82(100.0%)	
Gender	Male	61(72.6%)	23(27.4%)	84(100.0%)	(0.69)
	Female	46(69.7%)	20(30.3%)	65(100.0%)	
Smoking	Smoker	21(77.8%)	6(22.2%)	27(100.0%)	(0.41)
	Non-Smoker	86(69.9%)	37(30.1%)	143(100.0%)	
Severity of Disease	Mild – Moderate (SpO_2_ ≥ 90%)	26(100%)	0(0%)	26 (100.0%)	(0.00)*
	Severe (SpO_2_≤ 89%)	81(65.3%)	43(34.7%)	124 (100.0%)	
ARDS	Present	4(19%)	17(81%)	21(100.0%)	(0.00)*
	Absent	103(79.8%)	26(20.2%)	129(100.0%)	
Mechanical Ventilation	Assisted Ventilation	91(98.9%)	1(1.1%)	92(100.0%)	(0.00)*
	No Assisted Ventilation	16(27.6%)	42(72.4%)	58(100.0%)	
Comorbidities	Co-morbidity present	44(88.0%)	6(12.0%)	50(100.0%)	(0.01)*
	No comorbidity	63(63.0%)	37(37.0%)	100(100.0%)	
*Lab Parameters*					
Baseline pH	≤7.39	80(65%)	43(35%)	123(100.0%)	(0.00)*
	≥7.40	27(100%)	0(0%)	27(100.0%)	
Baseline PCO_2_	<35 mmHg	10(83.3%)	2(16.7%)	12(100.0%)	(0.09)
	35-45mmHg	13(92.9%)	1(7.1%)	14(100.0%)	
	>45mmHg	84(67.7%)	40(32.3%)	124(100.0%)	
Baseline P02	<75 mmHg	76(65%)	41(35%)	117(100.0%)	(0.001)*
	75-100 mmHg	31(93.9%)	2(6.1%)	33(100.0%)	
Baseline HCO_3_	<22 mEq/L	61(67.8%)	29(32.2%)	90(100.0%)	(0.30)
	22-26 mEq/L	24(82.8%)	5(17.2%)	29(100.0%)	
	>26 mEq/L	22(71.0%)	9(29.0%)	31(100.0%)	
Baseline Ferritin	11-336 mcg/L	9(69.2%)	4(30.8%)	13(100.0%)	(0.10)
	337-700 mcg/L	27(84.4%)	5(15.6%)	32(100.0%)	
	700-1000 mcg/L	38(76.0%)	12(24.0%)	50(100.0%)	
	1001-2000 mcg/L	32(61.5%)	20(38.5%)	52(100.0%)	
	>2000 mcg/L	1(33.3%)	2(66.7%)	3(100.0%)	
TLC	Normal Range (4-11.0 10^9/L)	36(83.7%)	7(16.3%)	43(100.0%)	(0.001)*
	High (11.1-15.0 10^9/L)	37(82.2%)	8(17.8%)	45(100.0%)	
	Very High (>15.0 10^9/L)	34(54.8%)	28(45.2%)	62(100.0%)	
LDH	Normal 100-300 IU/L	44(89.8%)	5(10.2%)	49(100.0%)	(0.00)*
	High >300 IU/L	63(62.4%)	38(37.6%)	101(100.0%)	
CRP	Normal <10 mg/L	4(80.0%)	1(20.0%)	5(100.0%)	(0.66)
	High > 10 mg/L	103(71.0%)	42(29.0%)	145(100.0%)	
D-dimer	Normal <0.5 mcg/mL	14(82.4%)	3(17.6%)	17(100.0%)	(0.28)
	Positive > 0.5 mcg/mL	93(69.9%)	40(30.1%)	133(100.0%)	

*Abbreviations:* p stands for probability of rejecting null hypothesis when it is true. *Footnotes:* Statistical significance for difference in proportions are calculated using Pearson’s Chi-Squared test. P less than 0.05 was considered statistically significant, Percentages are Row wise.

Having ARDS and need of assisted mechanical ventilation is closely related to outcome as well as depicted again by significant *p-value* <0.05. To test whether age, gender, BMI, SpO_2_, CO_2_, HCO_3_, Ferritin, LDH, CRP, TLC, D-dimer and comorbid conditions predict the outcome of patient in terms of cure or mortality, logistic regression was performed.

### Note:

Dependent variable: Outcome. Predictor: age, gender, BMI, SpO_2_, PCO_2_, HCO_3_, Ferritin, LDH, CRP, TLC, D-dimer and comorbidities, n=150, ^*^ =p<0.05, ^* *^ =p<0.01

The results of logistic regression conducted to predict outcome based on age (mean=59.73, SD=11.35), BMI (mean=30.14, SD=7.30), SpO_2_ (mean=80.66, SD=9.15), PCO_2_ (mean=58.46, SD=18.04), HCO_3_ (mean=21.8, SD=5.75), TLC(mean=14.56, SD=5.68), Ferritin (mean=920.28, SD=403.70), LDH (mean=508.09, SD=315.06), D-dimer (mean=14.98, SD=90.15), CRP (mean=121, SD=68.49) are shown in Table-III.

**Table-III:** Logistic Regression table of age, gender, BMI, SpO_2_, C O_2_, HCO_3,_ Ferritin, LDH, CRP, TLC, D-dimer and comorbid conditions predicting outcome.

Variables	B	S. E	Wald	df	Sig.	Exp(B)	95% C.I. for EXP(B)	
							Lower	Upper
Age	.021	.025	.727	1	.394	1.021	.973	1.072
BMI	.092	.043	4.670	1	.031*	1.096	1.009	1.192
Gender	.215	.556	.149	1	.700	1.239	.417	3.686
SpO_2_	-.158	.043	13.657	1	.000*	.854	.785	.929
PCO_2_	.002	.018	.014	1	.907	1.002	.967	1.039
HCO_3_	-.109	.049	4.957	1	.026*	.897	.815	.987
TLC	.077	.050	2.395	1	.122	1.080	.980	1.190
Ferritin	-.001	.001	1.035	1	.309	.999	.998	1.001
LDH	.002	.001	3.175	1	.075	1.002	1.000	1.004
d-Dimers	.018	.030	.355	1	.551	1.018	.959	1.081
CRP	.005	.004	1.674	1	.196	1.005	.997	1.013
Co-Morbidity (1)	-.350	.689	.257	1	.612	.705	.182	2.722
Constant	7.170	3.996	3.219	1	.073	1299.208		

The logistic regression model was found to be significant (*x^2^* (12) =75.406, *p*<0.000). A large amount of variance is explained in outcome due to predictors (R2=0.566). Significant odd ratios exist for BMI (Wald (1) =4.670, *p*=0.031, OR=1.096), SpO_2_ (Wald (1) =13.657, *p*=0.000, OR=0.854), HCO_3_ (Wald (1) =4.957, *p*=0.026, OR=0.897). Results suggest that increase in BMI increase the likelihood of mortality while decreasing SpO_2_ and HCO_3_ will also increase the chances of mortality.

## DISCUSSION

This study probes the intricate connection between obesity and COVID-19 outcomes, shedding light on the interaction between infectious illnesses and host variables, particularly in critical care. Our study found that 82(54.7%) of COVID-19 patients admitted to the ICU had a BMI above 30, and obesity was significantly associated with increased mortality 33(76.7%). Among the obese patients, 47% required invasive mechanical ventilation. Comparatively, a study conducted in France by Caussy C et al.^10^ reported that among 306 ICU patients without COVID-19, 26% had obesity, while 47.6% of critical COVID-19 patients had obesity, which was 47.6% higher than non-COVID-19 ICU patients (p < 0.0001). They also found that the odds ratio (OR) for obesity among COVID-19 ICU patients was 2.86 (95% CI 1.78–4.61) stressing the increased vulnerability of obese patients. These findings strengthen the global observation, that obesity is a significant risk factor for adverse outcomes in COVID-19 patients.

In comparison to our findings, a multi-center retrospective cohort study by Chetboun M et al.^11^ showed a linear association between BMI and the need for invasive mechanical ventilation in critically ill COVID-19 patients. This was independent of other metabolic risk factors. In our study, we observed a significant correlation between elevated BMI and increased mortality rates, with 54.7% of obese patients succumbing to the disease. Our results align with the global evidence that obesity is a major risk factor for both severe disease and mortality in COVID-19 patients.

In another study done by Sharon Viscardi included two hundred patients that are almost similar to our sample size (n=150) He showed that obesity is significantly associated with poor outcomes in COVID-19 patients, including prolonged hospital stays, higher incidence of ARDS, and higher mortality rates and these patients have an increased risk of requiring mechanical ventilation.^12^ Like in our study mortality was higher (40.2%) in obese vs. (14.7%) in non-obese patients. Yet another study analyzed data from 300 COVID-19 patients at King Abdulaziz University Hospital in Saudi Arabia. Mortality was significantly higher among obese patients compared to overweight patients (10.4% vs. 3.8%), He also showed intubation rate was higher in obese patients compared to overweight (34.6% vs. 22.7%), concluding that obesity is a clinically important predictor for severe COVID-19 leading to pneumonia that may require intubation. 13

Parianos M studied the effect of obesity on COVID-19 outcomes among hospitalized older adults in 2022. According to him, obesity is a modifiable risk factor that negatively affects COVID-19 outcomes.^14^ Likewise it is very true in our case as well. Mohamed Nakeshband^15^ described that patients with high BMI with COVID-19 are at a greater risk for death. As there was significantly increased risk of mortality in the overweight (RR 1.4, 95% CI 1.1-1.9) and obese groups (RR 1.3, 95% CI 1.0-1.7) compared with those with normal BMI. This study showed that obesity appears to significantly upsurge the risk of mortality in males (RR 1.4, 95% CI 1.0-2.0, P = 0.03) compared to females (RR 1.2, 95% CI 0.77-1.9, P = 0.40). However, in our study mortality was almost same for both (males: 27.4%, females: 30.3%). Likewise, the multicenter study by Chetboun M et al.^11^reported a median BMI of 28.1 kg/m², with 73.9% of critically ill COVID-19 patients requiring invasive mechanical ventilation (IMV) and a 28-day mortality rate of 36.1%. They found a linear association between BMI and IMV (OR 1.27 per 5 kg/m²) and higher mortality in obesity class III (HR 1.68). In our study, elevated inflammatory markers, including CRP, ferritin, and D-dimer, were significantly associated with severe COVID-19 outcomes. Specifically, patients with severe ARDS exhibited notably higher levels of these markers compared to those without ARDS, further supporting their role in predicting disease severity. Similar findings were reported by Zeng F et al.^16^, who conducted a meta-analysis of 16 studies involving 3,962 patients. Their analysis demonstrated that patients in the non-severe group had significantly lower levels of CRP (WMD = −41.78 mg/l), serum ferritin (WMD = −398.80 mg/l), IL-6 (WMD = −21.32 ng/l), and other inflammatory markers compared to the severe group. These differences highlight the potential of these biomarkers as predictors of disease progression. Additionally, Zeng et al. found that survivors had lower IL-6 levels than non-survivors (WMD = −4.80 ng/ml), a finding consistent with the higher inflammatory burden observed in our patients who suffered adverse outcomes, including mortality.

In abridgment, our study accentuates obesity as a crucial and modifiable risk factor for adverse COVID-19 outcomes. The evidence underscores the need for targeted interventions in risk assessment and resource allocation. Collaborative efforts between researchers, policymakers, and healthcare practitioners are essential for effective interventions addressing the complex interplay of host variables, infectious diseases, and clinical outcomes.

### Limitation

The study was conducted at a single centre, which may limit the generalizability of our findings. Secondly, the sample size, while adequate for initial analysis, still may not be representative of broader population. Thirdly, there may be certain potential confounding factors like comorbidities that remained undiagnosed and socioeconomic factors that do impact patient outcome we were unable to account for such factors. Finally, the observational nature of the study restricts the ability to establish definite cause between obesity and COVID-19 severity. Future studies with larger, more diverse populations and more comprehensive data collection could address these limitations.

## CONCLUSION

Our study highlights a strong connection between obesity and severe outcomes in COVID-19 patients. We found that obesity significantly worsens disease severity and increases mortality risk, suggesting the need for better risk assessment. This research underscores the importance of recognizing obesity as a major factor in infectious diseases, prompting a shift in how we approach healthcare in the post-COVID era. By broadening our focus to include other contributing factors, we aim to plan strategies that will strengthen global health systems, making them more resilient in future pandemics.

### Authors Contribution:

**MI & AR:** Conceived, designed, statistical analysis & manuscript Writing

**SZ:** Data collection, literature review, manuscript Writing.

**AN:** Review, editing & help in statistical analysis.

All authors have read the final version of the manuscript and are responsible for the accuracy or integrity of the work.
